# Combinatorial Antimicrobial Efficacy and Mechanism of Linalool Against Clinically Relevant *Klebsiella pneumoniae*

**DOI:** 10.3389/fmicb.2021.635016

**Published:** 2021-03-17

**Authors:** Shun-Kai Yang, Khatijah Yusoff, Mokrish Ajat, Chien-Yeong Wee, Polly-Soo-Xi Yap, Swee-Hua-Erin Lim, Kok-Song Lai

**Affiliations:** ^1^Department of Cell and Molecular Biology, Faculty of Biotechnology and Biomolecular Sciences, Universiti Putra Malaysia, Serdang, Malaysia; ^2^Department of Microbiology, Faculty of Biotechnology and Biomolecular Sciences, Universiti Putra Malaysia, Serdang, Malaysia; ^3^UPM-MAKNA Cancer Research Laboratory, Institute of Bioscience, Universiti Putra Malaysia, Serdang, Malaysia; ^4^Department of Veterinary Preclinical Sciences, Faculty of Veterinary Medicine, Universiti Putra Malaysia, Serdang, Malaysia; ^5^Biotechnology and Nanotechnology Research Centre, Malaysian Agricultural Research and Development Institute (MARDI), Kuala Lumpur, Malaysia; ^6^Department of Medical Microbiology, Faculty of Medicine, University of Malaya, Kuala Lumpur, Malaysia; ^7^Health Sciences Division, Abu Dhabi Women’s College, Higher Colleges of Technology, Abu Dhabi, United Arab Emirates

**Keywords:** adjuvant–antibiotic combinatory therapy, carbapenemase-producing *Klebsiella pneumoniae*, linalool, membrane disruption, oxidative stress

## Abstract

Antibiotic–adjuvant combinatory therapy serves as a viable treatment option in addressing antibiotic resistance in the clinical setting. This study was carried out to assess and characterize the adjuvant potential and mode of action of linalool against carbapenemase-producing *Klebsiella pneumoniae* (KPC-KP). Linalool exhibited bactericidal activity alone (11,250 μg/ml) and in combination with meropenem (5,625 μg/ml). Comparative proteomic analysis showed significant reduction in the number of cytoplasmic and membrane proteins, indicating membrane damage in linalool-treated KPC-KP cells. Upregulation of oxidative stress regulator proteins and downregulation of oxidative stress-sensitive proteins indicated oxidative stress. Zeta potential measurement and outer membrane permeability assay revealed that linalool increases the bacterial surface charge as well as the membrane permeability. Intracellular leakage of nucleic acid and proteins was detected upon linalool treatment. Scanning and transmission electron microscopies further revealed the breakage of bacterial membrane and loss of intracellular materials. Linalool induced oxidative stress by generating reactive oxygen species (ROS) which initiates lipid peroxidation, leading to damage of the bacterial membrane. This leads to intracellular leakage, eventually killing the KPC-KP cells. Our study demonstrated that linalool possesses great potential in future clinical applications as an adjuvant along with existing antibiotics attributed to their ability in disrupting the bacterial membrane by inducing oxidative stress. This facilitates the uptake of antibiotics into the bacterial cells, enhancing bacterial killing.

## Introduction

Antibiotic resistance in clinical settings has been a major public health concern, extending the treatment time while increasing mortality rates among the neonates, elderly, and immunocompromised (Ventola, 2015). Antibiotic resistance in bacteria can be acquired over time, from genetic mutations to horizontal gene transfer, *via* inter- and/or intraspecies. One common mechanism of antibiotic resistance involves the production of a variety of beta lactamases which essentially deactivates beta lactam antibiotics (Yap et al., 2017; Yang et al., 2018). Some bacteria are also able to reduce their membrane permeability either by reducing the expression of channel proteins on their membrane or the expression of efflux which rapidly removes the antibiotics that had penetrated the cell membrane (Yang et al., 2018). It is due to the combination of mechanisms mentioned above that will enable the bacteria to overcome antibiotic treatments and still survive. *Klebsiella pneumoniae*, a Gram-negative bacilli, is an opportunistic bacteria in clinical settings and is capable of causing nosocomial infections such as urinary, respiratory, and bloodstream infections, especially in intensive care unit patients (Paczosa and Mecsas, 2016). In recent decades, the debut of carbapenemase-producing *K. pneumoniae* (KPC-KP) had reduced the efficacy of antibiotic therapy. KPC-KP cells was first isolated from a patient in the National Institutes of Health Clinical Center, New York City (Snitkin et al., 2012). The KPC-KP cells eventually infected 17 other patients whereby 10 were killed in the course of the infections (Snitkin et al., 2012). The isolates were resistant to all tested antibiotics, including carbapenemase, gentamicin, tigecycline, and colistin. In addition, studies on the KPC-KP cells revealed that they had permeability defects which typically reduce the uptake of antibiotics due to reduced expression of protein channels (Snitkin et al., 2012). Another study had reported enhanced expression of multidrug resistance efflux pump in the presence of antibiotics (Snitkin et al., 2012). Taken together, these mechanisms effectively confer high resistance of KPC-KP cells toward antibiotics.

The ability to alter bacterial membrane permeability plays a vital role in KPC-KP cells in avoiding death from antibiotic treatment. To address this issue, combinatory therapy can be applied in patients during infection. Traditionally, antibiotic combinatory therapy consisted either of an antibiotic–antibiotic combination or antibiotic–adjuvant combination (Moo et al., 2019). Antibiotic–antibiotic combination refers to the use of two antibiotics of different classes and modes of action; this was known to reduce the development of antibiotic resistance to either of the antibiotics which enhances killing of the bacterial cells (Moo et al., 2019). For instance, the colistin–tigecycline combo is an antibiotic–antibiotic combination of different classes with different modes of action and had been used previously against carbapenemase-producing *Enterobacteriaceae*, while the amoxicillin–clavulanic acid combo is an antibiotic–adjuvant combination whereby clavulanic acid deactivates beta lactamase enzyme produced by the bacteria and, thus, makes the bacteria susceptible to amoxicillin infection (Tängdén, 2014). The antibiotic–adjuvant combo refers to the combination of an antibiotic and an adjuvant; the adjuvant functions to deactivate the resistant mechanism of the bacterial cells (Douafer et al., 2019). Antibiotic–adjuvant therapy can be categorized into three separate groups: the first group consists of adjuvants that have very low antibacterial activity but which enhance the antibiotic actions, group 2 consists of auxiliary compounds which support the antibiotic activity by affecting the efflux pump or altering the membrane permeability of the bacterial cell, while group 3 directly affects the physiological activity of the bacterial cells (Douafer et al., 2019). The application of group 1 adjuvant had long been established in clinical settings, yet resistance had been observed throughout time. Resistant bacterial cells treated by a combination of antibiotic and group 1 adjuvant such as clavulanic acid had been observed to resist clavulanic acid by overexpressing the beta lactamase enzyme which neutralizes the effect of clavulanic acid (Easton et al., 2003). Information regarding group 2 adjuvants are limited and are currently being studied for their usage in clinical settings. A study by Amaral et al. (2007) reviewed enhanced killing of *Mycobacterium tuberculosis* by a combination of antibiotics with several auxiliary compounds such as phenothiazine, chlorpromazine, and thioridazine which disrupt the efflux activity of the bacteria (Amaral et al., 2007). This showed the potential of membrane-permeabilizing compounds in counteracting the antibiotic resistance in clinical settings. Group 3 adjuvants include efflux pump inhibitors such as conessine and 4-isopentyloxy-2-naphthamide which directly block bacterial efflux pumps, facilitating the uptake while preventing the removal of antibiotic from within the bacterial cell (Whalen et al., 2015; Siriyong et al., 2017).

Linalool is a natural plant secondary metabolite belonging to the acyclic monoterpene alcohol group with the molecular formula of C_1__0_H_1__8_O (Elisabetsky, 2002). It is commonly found as a major component of essential oils such as lavender, basil, etc. Linalool has been heavily used in the fragrance and cosmetic industries (Mahizan et al., 2019). Studies have shown that linalool exhibited antibacterial activity against a panel of bacteria such as *Escherichia coli*, *Pseudomonas aeruginosa*, *Salmonella typhimurium*, and *Staphylococcus aureus* (Herman et al., 2016; Prakash et al., 2019). Our previous study revealed that linalool is one of the major components found in lavender essential oil, and might be responsible in the bactericidal activity of KPC-KP cells (Yang et al., 2019b). However, the mode of action of linalool has yet to be fully understood. Therefore, this study was performed to determine the combinatory effect of linalool and meropenem against KPC-KPC cells and to understand its fundamental mode of action using comparative proteomic profiling. In addition, both quantitative and qualitative assays were used to elucidate the underlying mechanism of linalool. Taken together, the study served as an important foundation in future applications of linalool as an adjuvant in adjuvant–antibiotic combinatory therapy.

## Materials and Methods

### Linalool and Meropenem

Linalool (purity ≥ 97%) used in this study was purchased from MERCK (New Jersey, United States), while meropenem was from Sigma-Aldrich Corporation (Missouri, United States). Linalool was dissolved in Mueller–Hinton broth from Sigma-Aldrich Corporation (Missouri, United States), supplemented with 10% Tween 80 to enhance solubility. Meropenem at a concentration of 10 mg/ml was disolved in sterile Milli-Q water to make a concentrated stock for subsequent experiments.

### Culture Condition of Bacterial Strain

KPC-KP (*K. pneumoniae* ATCC^®^ BAA-1705^TM^ carrying the *blaKPC* gene) was purchased from the ATCC (Virginia, United States). The bacterial strain was grown and maintained on Mueller–Hinton agar by Sigma-Aldrich Corporation (Missouri, United States) at 37°C for 18 h. The experimental bacterial culture was grown from a single colony of KPC-KP cells in Mueller–Hinton broth at 37°C with shaking at 200 rpm for 18 h.

### Antimicrobial Susceptibility and Checkerboard Assay

Antimicrobial susceptibility assay was performed to determine the minimum inhibitory concentration (MIC) of linalool and meropenem against KPC-KP cells, as detailed in Yang et al. (2017). Two-fold dilutions were performed on linalool and meropenem in their respective wells to yield a final composition of 50 μl of linalool or meropenem, 40 μl of KPC-KP cells at a final concentration of 1 × 10^5^ CFU/ml, and 10 μl of resazurin from Sigma-Aldrich Corporation (Missouri, United States) at a final concentration of 0.02% (w/v). The concentration range of linalool was 45,000–1,406 μg/ml, while meropenem was 128–8 μg/ml. The experiments included growth control (KPC-KP cells only), sterility control (Mueller–Hinton broth), and antibiotic-positive control (*E. coli* ATCC 25922).

The checkerboard assay was performed to determine the combinatory activity of linalool and meropenem and is detailed in Yang et al. (2017). Ten serial, two-fold dilutions of meropenem and five serial, two-fold dilutions of linalool were prepared, and each well contained 25 μl of meropenem and 25 μl of linalool, inoculated with 40 μl of KPC-KP cells to make a final concentration of approximately 1 × 10^5^ CFU/ml and 10 μl of resazurin to make a final concentration of 0.02%. The 96-well plates were then incubated at 37°C with shaking at 200 rpm for 20 h. The assay was completed in triplicate. The combinatory relationship between linalool and meropenem was expressed in terms of combined fractional inhibitory concentration index (FICIc) using the following formulae (Yang et al., 2017):

$$F\,I\,C\,I\,o\,f\,l\,i\,n\,a\,l\,o\,o\,l=\frac{M\,I\,C\,o\,f\,l\,i\,n\,a\,l\,o\,o\,l\,i\,n\,c\,o\,m\,b\,i\,n\,a\,t\,i\,o\,n}{M\,I\,C\,o\,f\,l\,i\,n\,a\,l\,o\,o\,l\,a\,l\,o\,n\,e}$$

$$F\,I\,C\,I\,o\,f\,m\,e\,r\,o\,p\,e\,n\,e\,m=\frac{M\,I\,C\,o\,f\,m\,e\,r\,o\,p\,e\,n\,e\,m\,i\,n\,c\,o\,m\,b\,i\,n\,a\,t\,i\,o\,n}{M\,I\,C\,o\,f\,m\,e\,r\,o\,p\,e\,n\,e\,m\,a\,l\,o\,n\,e}$$

F⁢I⁢C⁢I⁢c=F⁢I⁢C⁢I⁢o⁢f⁢l⁢i⁢n⁢a⁢l⁢o⁢o⁢l+F⁢I⁢C⁢I⁢o⁢f⁢m⁢e⁢r⁢o⁢p⁢e⁢n⁢e⁢m

FICIc ≤ 0.5, synergistic; FICIc > 0.5–4.0, additive; FICIc > 4.0, antagonistic.

The experiment was performed in triplicate and incubation was performed at 37°C with shaking at 200 rpm for 18 h.

### Time Kill Assay

The final concentration of linalool and meropenem was as determined in the Antimicrobial Susceptibility and Checkerboard Assay section, while 1 × 10^5^ CFU/ml of KPC-KP cells were introduced in the treatment culture. The assay consisted of the nontreated KPC-KP cells (innoculum in MHB supplemented with 10% Tween 80), linalool-treated KPC-KP cells, meropenem-treated KPC-KP cells, and linalool–meropenem-treated KPC-KP cells. Cells were incubated at 37°C with shaking at 200 rpm. Viable counting was performed immediately upon inoculation and every 30 min for 4 h by plating 20 μl of culture media onto Mueller–Hinton agar as determined in our previous study based on lavender essential oil. From our previous study, the combination of lavender essential oil and meropenem completely killed KPC-KP cells in 1.5 h. Considering that the major compositions of lavender essential were linalool (34.5%) and linalyl anthranilate (45.9%) and the doubling time of KPC-KP cells is approximately 30 min, a 4-h killing curve is more than enough to produce any visible growth. Plated cells were incubated at 37°C for 18 h. The experiment was performed in triplicate.

### Comparative Proteomic Analysis *via* Nano-Liquid Chromatography Tandem Mass Spectrophotometry

Protein extraction was first performed as detailed in Yang et al. (2019a). The protein concentration of each sample was standardized to 1 mg/ml for subsequent proteomic analysis. Approximately 100 μg of total protein was resuspended in 100 μl of 50 mM ammonium bicarbonate (pH 8.0). A RapiGest surfactant (Waters Corporation, United States) was added to the extracted protein in equal parts of 100 μl at a final concentration of 0.05%. The proteins from each sample were then concentrated to a volume of 100 μl using a Vivaspin^TM^ column (GE Healthcare, United States) with a molecular weight cutoff (MWCO) of 3,000 kDa as per the manufacturer’s instruction. Protein samples were then digested using Trypsin Gold (Promega, United States) as per instruction from the manufacturer. The tryptic peptide solution from each sample was centrifuged at 14,000 rpm for 20 min, and the resulting supernatants were collected and kept at −80°C until subsequent analysis.

Nano-liquid chromatography tandem mass spectrophotometry (nano-LC-MS/MS) analysis was performed as detailed in Yang et al. (2019a) using an Orbitrap Fusion Tribrid mass spectrometer (Thermo Fisher Scientific, United States). The Thermo Scientific^TM^ Proteome Discoverer^TM^ Software v2.1 with the SEQUEST^®^ HT search engine was used to process the obtained raw data from all protein samples. The MS ion intensities were calculated based on the accurate mass and time tag strategy. The accurate alignment of detected LC retention time and *m*/*z* value across different analyses and the area under chromatographic elution profiles of the identified peptides can be compared between different samples. For protein identification, the peptide identification data was compared with the UniProt^®^ *K. pneumoniae* database with a 1% strict FDR and 5% relax FDR criteria using Percolator^®^. Search parameters were set up to two miscleavages with fixed amino acid modification through carbamidomethylation and variable modification through methionine oxidation, together with asparagine and glutamine deamidation. A fragment tolerance of 0.6 Da and a precursor tolerance of 10 ppm were used with trypsin as a digestion enzyme. The identified proteins with at least two unique peptides implied a greater confidence of protein identity. Protein quantification and statistical analyses were performed using Perseus software v1.6.0.7 (Max Planck Institute of Biochemistry). Each control and treated sample consisted of three biological replicates with three technical replicates, each analyzed by LC-MS/MS. The protein file with three technical replicates in txt. format from the Proteome Discoverer^TM^ was uploaded to the Perseus system for further comparative analysis between samples. The data were log2-transformed to stabilize the variance and scale-normalized to the same mean intensity across the technical replicates. The mean values for all three technical replicates of the same biological samples were grouped together in the same matrix, and valid values were obtained by filtering with “at least two,” eliminating proteins, which only existed in one of the technical replicates. Finally, all biological replicates of the same treatment group were consolidated under the same matrix, with the missing values imputed with the random numbers that are drawn from a normal distribution. The histograms were plotted to get a profile for similarity comparison of the ratio for all the samples. Differentially expressed proteins between control and treatment were detected using *t*-test, and the *p*-values were also adjusted for multiple testing using the permutation-based false discovery rate, with a number of randomization of 250. Proteins were considered to be significantly differentially expressed between treatment groups with an adjusted *q* value < 0.05 and a fold change ≤ −1 or ≥ 1.

### Zeta Potential Measurement

Cell treatments were detailed in the *Time Kill Assay* section. The zeta potential of nontreated, linalool-treated, and meropenem-treated KPC-KP cells was measured with a Zetasizer Nano ZS instrument (Malvern Instruments, Malvern, United Kingdom). The treatment time for all the treatment groups was as determined in the time kill analysis, whereas the concentrations of linalool and meropenem used were as determined from the checkerboard assay. Treated cells were washed with 0.85% saline at least five times before zeta potential measurement. The experiment was performed in triplicate.

### Outer Membrane Permeability Assay

Outer membrane permeability assay was performed as detailed in Yang et al. (2017). KPC-KP cells with optical density (OD600 nm) of 0.3 were treated with linalool as shown in the *Time Kill Assay* section. After treatment, samples were washed with 0.85% saline and divided into two equal portions of 10 ml. Portion 1 was treated with sodium dodecyl sulfate (SDS) solution at a final concentration of 0.1% and portion 2 was treated with 0.85% saline. SDS acts as a permeabilizing probe that causes cell death when a sudden influx occurred which was measured in terms of OD600 nm at intervals of 0, 5, 10, 30, and 60 min *via* a spectrophotometer. The assay was performed in triplicate.

### Nucleic Acid and Protein Leakage Measurent

Nucleic acid and protein leakage was measured as detailed in Yao et al. (2014). Cell treatment was as detailed in the *Time Kill Assay* section. Cell pellets of nontreated and linalool-treated KPC-KP cells were collected *via* centrifugation at 10,000 rpm for 5 min. The supernatant was collected and measured for nucleic acid at UV absorption wavelength of 260 nm and proteins using Bradford solution at UV absorption wavelength of 595 nm. The assay was performed in triplicate.

### Scanning and Transmission Electron Microscopy

Scanning electron microscopy (SEM) was performed as detailed in Yang et al. (2017). Cell treatment was as detailed in the *Time Kill Assay* section. Cell pellets were collected and washed with 0.85% (w/v) saline. Samples were then fixed using 4% glutaraldehyde for 5 h and 1% osmium tetroxide for 2 h at 4°C. Sodium cacodylate buffer at 0.1 M was used in all the subsequent washing steps. Samples were further dehydrated *via* sequential exposure to increasing concentrations of acetone (35–95%) for 10 min followed by 100% acetone for 15 min for three times. Following dehydration, samples were subjected to critical point drying for 30 min (Bal-Tec CPD 030, Bal-Tec, Balzers, Liechtenstein). Samples were secured onto the specimen stub using a double-sided tape, sputter-coated with gold using a cool sputter coater (Bal-Tec SCD 005), and observed *via* a JEOL JSM-6400 instrument (JEOL, Tokyo, Japan) at 15 kV.

For transmission electron microscopy (TEM), cell treatment was mentioned above. Cell pellets were collected and washed with 0.85% (w/v) saline, followed by fixation in 4% glutaraldehyde for 2 days at 4°C. Specimens were washed with 0.1 M sodium cacodylate buffer for 30 min for three times and postfixed in 1% osmium tetroxide for 2 h at 4°C. The specimen was then washed again for three times with 0.1 M sodium cacodylate buffer for 30 min. Specimens were then subjected to a series of acetone wash for dehydration purposes: with 35, 50, 75, and 95% acetone for 45 min in each washing. Pure acetone was used in the final washing step for 1 h and this was repeated for three times. Next, the specimens were infiltrated with various ratios of acetone and resin mixtures (1:1 for 12 h, 1:3 for 12 h, and 0:1 for 16 h), prior to beam capsulation. The capsulated specimens were polymerized at 60°C for 48 h. Polymerized specimen blocks were cut into 1-μm-thick sections and stained with toluidine blue and dried on a hot plate. The specimens were then viewed under a light microscope and the areas of interest were selected for ultrathin sectioning. Specimen blocks were trimmed according to selected area and ultrathin-sectioned using i-Ultramicrotome EM UC6 (Leica, Germany). Specimens were collected from the surface of the water bath and set on the copper meshes followed by uranyl acetate staining for 15 min and lead staining for 10 min. Samples were then viewed under the transmission electron microscope Leo LIBRA 120 (ZEISS, Germany).

### Lipid Peroxidation Assay

Lipid peroxidation assay was performed as detailed in Kumar et al. (2013). Cell treatment was as detailed in the *Time Kill Assay* section. Cell pellets of nontreated and linalool-treated KPC-KP cells were collected using centrifugation at 10,000 rpm for 5 min and washed with phosphate-buffered saline (PBS). The supernatant collected was termed treatment media. Collected cell pellets were then sonicated acording to the *Comparative Proteomic Analysis via Nano-Liquid Chromatography Tandem Mass Spectrophotometry* section. The supernatant obtained after sonication and centrifugation was collected and termed cell lysate. A volume of 500 μl of either treatment media or cell lysate was added to 400 μl of 15% trichloroacetic acid and 800 μl of 0.67% thiobarbituric acid (TBA) in 0.01% butylated hydroxytoluene. Samples were then vortexed and incubated at 95°C for 20 min in a water bath followed by the addition of 3 ml of butanol with gentle mixing. A volume of 200 μl of the butanol phase was collected from each sample with absorbance measured at 532 nm. The amount of maliodialdehyde (MDA) present was estimated using MDA standard curve and normalized based on the protein concentration of each sample. The assay was performed in triplicate.

### Reactive Oxygen Species Measurement

Reactive oxygen species (ROS) measurement was performed as detailed in Kumar et al. (2013). Cell treatment was as detailed in the *Time Kill Assay* section. The cell pellets of nontreated and linalool-treated KPC-KP cells were collected using centrifugation at 10,000 rpm for 5 min and washed with PBS. The cells were then treated with 20 μM of 2′,7′-dichlorofluorescein diacetate (DCF-DA) for 30 min at 37°C. The cell pellet was collected again using centrifugation at 10,000 rpm for 5 min with the supernatant consisting of DCF-DA removed. Cell pellets were resuspended in PBS and fluorescence intensity was measured at excitation and emission wavelengths of 485 and 528 nm using Tecan microplate reader (Tecan Trading AG, Switzerland). The assay was performed in triplicate.

## Results and Discussion

### Antimicrobial Activity and Combinatory Interaction Between Linalool and Meropenem Against KPC-KP Cells

Several of the reported studies performed have shown that linalool, a major component in a few essential oils, exhibited antibacterial activity against a panel of bacteria (Herman et al., 2016; Prakash et al., 2019). However, this has yet to be tested in multidrug-resistant strains. This study uses carbapenemase-producing *K. pneumoniae* which is known for their extensive resistance toward multiple classes of antibiotics, especially beta lactam antibiotics. The antimicrobial susceptibility assay revealed that linalool exerts antibacterial activity against KPC-KP cells at 11,250 μg/ml. Meropenem, one of the last resort antibiotics, is bactericidal to KPC-KP cells at 32 μg/ml, which is a very high concentration in comparison with the CLSI standards at 4 μg/ml against Enterobactericeae (Humphries et al., 2018). The combinatory interaction of linalool and meropenem significantly reduces the antibacterial dose by two-folds: 5,625 μg/ml for linalool and 16 μg/ml for meropenem Table 1).

**TABLE 1:** Minimum inhibitory concentration (MIC), fractional inhibitory concentration (FIC), and FIC indices of linalool and meropenem against KPC-KP cells.

**Combinations of linalool and meropenem (μg/ml)**	**KPC-KP**				**Type of interaction**
	**MIC_*O*_**	**FIC**	**FICI**	**FICIc**	
Linalool	11,250	5,625	0.50	1.00	Additive
Meropenem	32.00	16.00	0.50		

**MIC_*O*_, MIC of one component alone; FIC, MIC of one component in the most effective combination; FICI, FIC index of one component in the most effective combination; FICIc, total FICI of the combination of both components. FICIc ≤ 0.5, synergistic; FICIc > 0.5–4.0, additive; FICIc > 4.0, antagonistic.**

**FIGURE 1:**
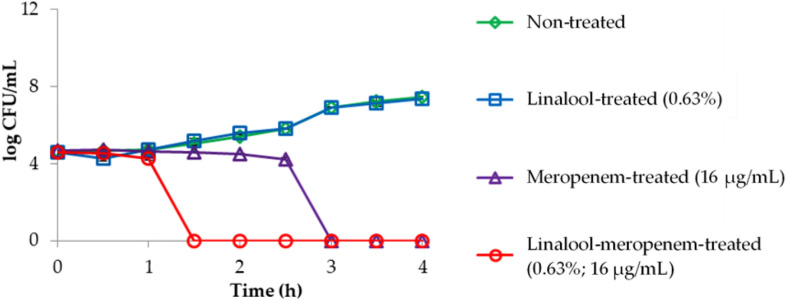
Time kill kinetics of KPC-KP cells treated with linalool (5,625 μg/ml) and meropenem (16 μg/ml), alone and in combination every 0.5 h for 4 h. LOD = 0.3 log CFU/ml.

The killing kinetics of linalool and meropenem against KPC-KP cells (Figure 1) shows that linalool alone at subinhibitory concentration had no effect on KPC-KP cells while meropenem at subinhibitory concentration inhibited the growth of KPC-KP cells within 3 h. In combination, the inhibitory time had been significantly shortened to 1.5 h. This demonstrated the potential of linalool as an adjuvant in combinatory treatment, by significantly lowering the effective dose of meropenem against KPC-KP cells while reducing treatment time.

### Proteomic Footprint of Bacterial Membrane Disruption Due to Oxidative Stress

**FIGURE 2:**
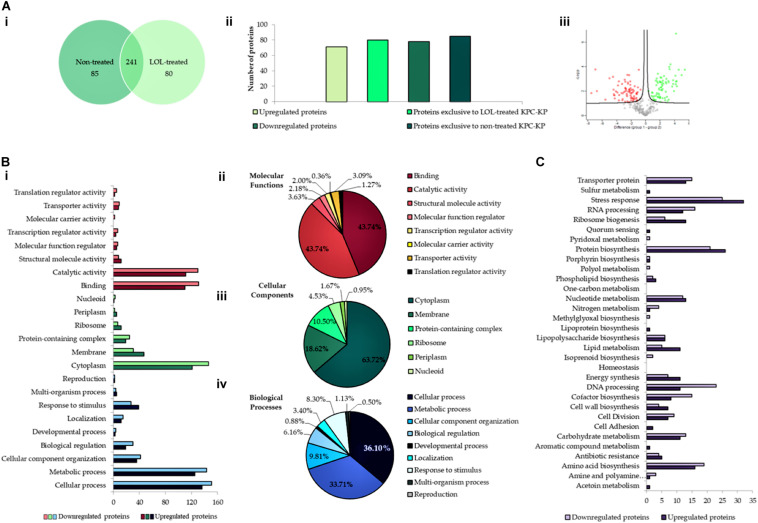
Comparative proteomic analysis between nontreated and linalool-treated KPC-KP cells. **(A-i)** Venn diagram of the total protein obtained from nontreated and linalool-treated KPC-KP cells. **(A-ii)** The total numbers of exclusive, upregulated and downregulated proteins. **(A-iii)** Volcano plot showing up- (designated green square) and downregulated (designated red square) proteins of the linalool-treated KPC-KP cells. Gene Ontology (GO) analysis of identified proteins and their relative abundance **(B-i)** in terms of molecular function **(B-ii)**, cellular components **(B-iii)**, and biological processes **(B-iv)** of linalool-treated KPC-KP cells. **(C)** KEGG pathway analysis of differentially expressed proteins in linalool-treated KPC-KP cells. The proteomic analysis is detailed in Supplementary Spreadsheet S1.

An extensive study on understanding the mode of action of linalool is required prior to their application as an antimicrobial adjuvant in the clinical setting. To date, no studies have reported the mode of action of linalool as an antibacterial agent. Previous studies on linalool-containing essential oils have postulated on their ability in disrupting the bacterial membrane (Yang et al., 2019b). However, the exact fundamental mechanism of linalool has not been properly investigated. In this study, comparative proteomic profiling was performed to elucidate the underlying mechanism of linalool against KPC-KP cells, by looking at the overall changes in the proteome of linalool-treated KPC-KP cells in comparison with nontreated KPC-KP cells. From the proteomic analysis, a total of 326 proteins were identified from the nontreated KPC-KP cells, whereas only 321 proteins were identified from the linalool-treated KPC-KP cells (Figure 2A). Both groups shared a total of 241 similar proteins. In addition, 71 proteins were upregulated and 78 proteins were downregulated after treatment (Figure 2A).

Gene Ontology analysis was performed, classifying proteins of significant difference into three main categories: biological processes, cellular components, and molecular functions according to their significant abundance (Figure 2B). KPC-KP cells treated with linalool had decreased abundance of cytoplasmic and membrane-related proteins after treatment with linalool; 146 cytoplasmic proteins and 31 membrane-related proteins showed decreased abundance following linalool treatment, as compared with the nontreated KPC-KP cells. This indicated a disrupted bacterial membrane which leads to intracellular leakage, explaining the loss of cytoplasmic proteins.

From the proteomic KEGG pathway analysis shown in Figure 2C, seven proteins related to stress response had increased in abundance following linalool treatment (Table 2). Data obtained from the proteomic analysis inferred that linalool induces oxidative stress in KPC-KP cells. Oxidative stress is known to affect the integrity of cell membranes, termed lipid peroxidation. Lipid peroxidation is a self-propagating chain reaction which involves reactions between a ROS and membrane fatty acid, eventually destroying the membrane of a cell (Wong-Ekkabut et al., 2007; Van der Paal et al., 2016). In addition, majority of the proteins involved in ribosome biogenesis and DNA and RNA processing had decreased abundance after linalool treatment, when compared with nontreated KPC-KP cells (Table 2). Generally, ribosomal and genetic material processing proteins are relatively sensitive to oxidative stress (Yang et al., 2019b). For instance, oxidative damage can induce base substitution, addition, deletion, and other mutations in nucleic acids which lead to the formation of non-functioning proteins (Guo et al., 2017). In addition, oxidative damage also affects proteins, especially ribosomal proteins due to the affinity of ROS for RNA (Kong and Lin, 2010). A previous study has shown that oxidation of RNA indirectly causes damage to ribosomal RNA, *via* covalent modification, leading to defective protein synthesis (Kong and Lin, 2010). Thus, a decrease in the abundance of these proteins further indicated the presence of oxidative stress in KPC-KP cells during linalool treatment.

**TABLE 2:** List of upregulated oxidative stress regulator proteins and downregulated oxidative stress-sensitive proteins identified from linalool-treated KPC-KP cells.

**Accession no.**	**Protein**	**Fold change**
**Upregulated oxidative stress regulator proteins**		
B5XXP0	NAD(P)H dehydrogenase (quinone)	2.95
A6T5N6	Chaperone protein HtpG	2.47
A6TCJ1	Autonomous glycyl radical cofactor	2.36
B5XZ37	ATP-dependent protease ATPase subunit HslU	2.35
A6TCM1	Protein GrpE	1.97
A6T4F4	Chaperone protein DnaK	1.24
**Downregulated oxidative stress-sensitive proteins**		
A6TG44	Ribosomal RNA small subunit methyltransferase G	–4.26
A6THY6	Ribosomal RNA small subunit methyltransferase C	–3.91
B5XNB5	30S ribosomal protein S4	–3.34
A6T4I7	Ribosomal RNA small subunit methyltransferase A	–2.61
A6TEW9	50S ribosomal protein L2	–2.42
A6TEU2	Ribosomal RNA small subunit methyltransferase B	–2.23
B5XQC8	50S ribosomal protein L35	–2.09
A6T766	Ribosomal RNA large subunit methyltransferase I	–2.00
A6THB4	50S ribosomal protein L9	–1.97
A6THB1	30S ribosomal protein S6	–1.65

### Linalool Induces Oxidative Stress Which Damages Bacterial Membrane of KPC-KP Cells

**FIGURE 3:**
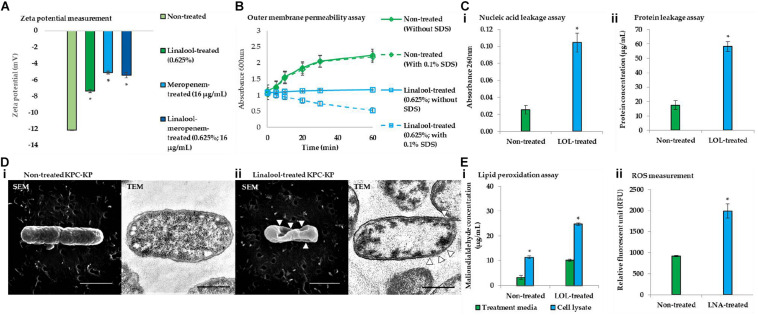
Linalool disrupts bacterial membrane of KPC-KP cells by inducing oxidative stress. **(A)** Zeta potential of non-treated, linalool-treated, and meropenem-treated KPC-KP cells, alone and in combination. **(B)** Outer membrane permeability of KPC-KP cells exposed to 0.1% SDS or saline after treatment with linalool at 5,625 μg/ml. **(C)** Intracellular leakage of UV-absorbing material: nucleic acid (i) and protein (ii) of KPC-KP cells exposed to linalool. **(D)** Scanning and transmission electron micrographs of non-treated (i) and linalool-treated (ii) KPC-KP cells (Δ indicates membrane damage). **(E)** Oxidative stress assessment *via* ROS measurement (i) and lipid peroxidation assay (ii). * indicates significant difference.

Membrane-related assay such as zeta potential measurement, outer membrane permeability assay, intracellular leakage assay, and scanning and transmission electron microscopy were performed to determine the ability of linalool in disrupting the bacterial membrane of KPC-KP cells. Induction of oxidative stress by linalool was also validated *via* the lipid peroxidation assay and ROS measurement. Zeta potential measures the outer membrane charges of a bacterial cells; Gram-negative bacteria normally has negative membrane charges due to the presence of lipopolysaccharide in the outer membrane of the cell. It has been found in previous reports that surface charge neutralization leads to altered membrane permeability (Halder et al., 2015). From Figure 3A, the zeta potential of the nontreated KPC-KP cells showed a negative value of −12.1 mV, while linalool-treated cells had significantly more positive values of −7.38. Meropenem-treated KPC-KP cells had zeta potential value of −5.3 mV, which is more positive than the non-treated and meropenem-treated only cells; this indicated a greater effect in disruption of the bacterial membrane by inhibiting the bacterial cell wall formation, eventually causing damage to bacterial membrane *via* osmotic stress (Papp-Wallace et al., 2011). Linalool at subinhibitory concentration possibly only disrupted the outer membrane without damaging the cell wall and, thus, had a lower zeta potential value in comparison with meropenem. The combination of linalool and meropenem against KPC-KP cells caused a slight increment of the negative charge of the cells to −5.43 mV as compared with the meropenem-treated cells. However, there is no significant difference when compared with the zeta potential of meropenem alone. This observation result indicated that the interaction between linalool and meropenem was additive and did not interfere with one another.

The outer membrane permeability assay (Figure 3B) compares the growth of the nontreated KPC-KP cells and linalool-treated KPC-KP cells in terms of absorbance at 600 nm between pre- and post-exposure to 0.1% SDS solution. The nontreated group showed normal growth in the absence or presence of SDS. However, linalool-treated KPC-KP cells, in the absence 0.1% SDS, had lower absorbance compared with those of non-treated cells, indicating that the subinhibitory concentration of linalool slowed the growth of the KPC-KP cells. A significant drop in the absorbance can be detected when 0.1% SDS was introduced in the linalool-treated KPC-KP cells, indicating the ability of linalool to cause influx of SDS into the cell, eventually leading to killing of the cell. This indicated that the increase in permeability facilitates the uptake of antibiotics, namely meropenem, explaining the reduced effective dosage of meropenem in the presence of linalool as shown in the checkerboard assay.

As shown in Figure 3D, intracellular leakage assays revealed that linalool-treated KPC-KP cells had a significantly higher absorbance value of nucleic acid and protein concentrations as compared with non-treated KPC-KP cells, which infers loss of nucleic acids and proteins to the extracellular region. This further supports the intracellular leakage *via* membrane disruption as observed in the proteomic analysis. The scanning electron micrograph further revealed distortion in the shape and irregularities on the surface of linalool-treated KPC-KP cells, as indicated by the arrows in Figure 3D-ii. Nontreated KPC-KP cells retained their rod shape with minimal distortion (Figure 3D-i). In transmission electron microscopy, negative staining was performed using uranyl acetate due to its high affinity with biomolecules such as proteins, lipid, and nucleic acid (Winey et al., 2014). The microscopy revealed membrane damage and breakage in linalool-treated KPC-KP cells, along with intracellular leakage indicated by the poorly stained intracellular region (Figure 3D-ii). Nontreated KPC-KP cells had intact membranes with intense staining of uranyl acetate in their intracellular region (Figure 3D-i). The observed membrane disruption ability of linalool provides greater access for antibiotics to penetrate into the cells, eventually killing it. This explains the reduction of effective dosage of antibiotics during combination treatment of linalool and meropenem.

From the proteomic analysis, data suggested that membrane disruption is caused by the presence of oxidative stress during linalool treatment. To further strengthen the data from proteomic analysis, lipid peroxidation assay and ROS measurement were performed. The concentrations of MDA quantified in linalool-treated KPC-KP cells are significantly higher than those in the nontreated cells, indicating the presence of lipid peroxidation of cells during linalool treatment (Figure 3E-i). Additionally, the level of ROS quantified in linalool-treated KPC-KP cells was also significantly higher than that in the nontreated cells (Figure 3E-ii), indicating a higher level of ROS generated in KPC-KP cells upon exposure to linalool. The generated ROS attacks the bacterial membrane, causing lipid peroxidation, which disrupts the bacterial membrane as observed in previous assays.

## Conclusion

**FIGURE 4:**
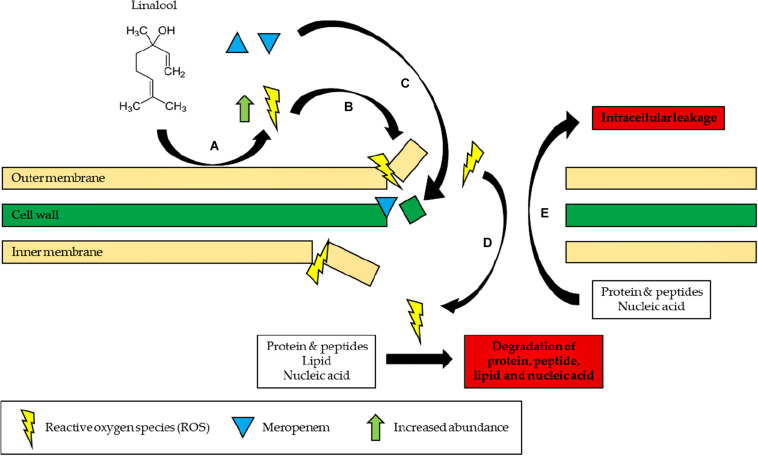
Proposed mode of action of linalool. **(A)** Linalool reacts with the bacterial outer membrane to form ROS. **(B)** ROS initiates lipid peroxidation and continuously damages the bacterial membrane. **(C)** Influx of meropenem into the cell, preventing cell wall synthesis. **(D)** Influx of ROS into the cell, degrading proteins, peptide, lipid, and nucleic acid by causing oxidative damage. **(E)** Proteins, peptide, lipid, and nucleic acid leak to the extracellular environment of the cells due to a disrupted membrane.

In conclusion, the study demonstrated that linalool could be used in antibiotic–adjuvant therapy as the subinhibitory concentration of linalool is sufficient to reduce the effective dosage of meropenem by two-folds in the resistant strain of *K. pneumoniae.* In addition, the mechanism of linalool has also been elucidated in this study whereby linalool disrupts the bacterial membrane by inducing oxidative stress as illustrated in Figure 4. The ability of linalool to disrupt bacterial membrane could significantly increase the uptake of antibiotics during therapy. This ensures that a lower dosage of antibiotics can be applied to negate the effect of antibiotic resistance. In addition, this might also revive the efficacy of previous antibiotics which can now be coupled in adjuvant–antibiotic combination therapy. Moving forward, a thorough cytotoxicity analysis will need to be carried out to determine the toxicity effects of linalool, especially in the animal model. This will expedite the application of linalool as an adjuvant in the clinical setting in the future.

## Data Availability Statement

The raw data supporting the conclusions of this article will be made available by the authors, without undue reservation.

## Author Contributions

K-SL, S-H-EL, and KY: conceptualization and methodology. C-YW: software. S-KY: validation, formal analysis, investigation, writing—original draft preparation, and visualization. C-YW, S-H-EL, and K-SL: resources. S-KY and K-SL: data curation. C-YW, MA, KY, P-S-XY, S-H-EL, and K-SL: writing—review and editing. S-H-EL and K-SL: supervision and project administration. S-H-EL and K-SL: funding acquisition. All authors contributed to the article and approved the submitted version.

## Conflict of Interest

The authors declare that the research was conducted in the absence of any commercial or financial relationships that could be construed as a potential conflict of interest.

## Supplementary Material

Supplementary Spreadsheet 1 Comparative proteomic analysis of non-treated and linalool-treated KPC-KP cells (Protein abundance, gene ontology analysis, and KEGG pathway analysis).
